# Extirpation of the Rectum for Carcinoma

**Published:** 1892-05

**Authors:** J. McFadden Gaston

**Affiliations:** Prof. Principles and Practice of Surgery, Southern Medical College, Atlanta, Ga.


ATLANTA
MEDICAL AND SURGICAL JOURNAL.

VOL. IX. MAY, 1892. No. 3.

EDITED BY
LUTHER B. GRANDY, M. D., AND MILLER B. HUTCHINS, M. D.,
WITH THE CO-OPERATION OF
H. V. M. MILLER, M. D., LL. D., VIRGIL O. HARDON, M. D.,
FLOYD W. McRAE, M. D., AND L. P. KENNEDY, M. D.

### *ORIGINAL COMMUNICATIONS.*

### EXTIRPATION OF THE RECTUM FOR CARCINOMA.*

By J. MACFADDEN GASTON, M. D.,
Prof. Principles and Practice of Surgery, Southern Medical College, Atlanta, Ga.

The patient referred to in the following clinical notes was presented at my office about the last of June by Dr. J. I. Darby, of Columbia, Ala.

He stated that some time previously he had treated the case for hemorrhoids by the injection of carbolic acid, with complete relief in that respect. But that subsequently there was trouble higher up in the rectum with more or less difficulty in evacuating the fæces, and that his general health had become impaired to some extent.

There was no family history of malignant disease, but his appearance led to the conclusion that there might be a cancerous cachexy. Having had occasion to report two cases of papilloma

*Read at the meeting of the Medical Association of Georgia, April 21st, 1892.

of the rectum with carcinomatous degeneration, which terminated fatally without operation, I recently advised in a similar case, under the care of Dr. K. C. Divine, the extirpation of the entire mass. This was done without serious consequences on May 30th, 1891, and promised a good result, but there was a redevelopment of the induration above with a fatal result.

Temporary relief has been afforded in one of my cases and in one of the above colleague by colostomy; but in this instance the radical operation seems to be indicated.

July 8th, 1891, Mr. W. C. E., a white man, sixty-two years old, apparently without any constitutional disorder, was received at the Providence Infirmary with rectal trouble. Upon digital examination and exploration with the speculum, there was found to exist an induration and thickening of the submucous tissues extending from an inch within the anus up to the recto-colic junction. The mucous membrane presented a dark congested appearance attended with some oozing of blood. There was a marked constriction of the upper portion of the indurated structure, but admitting of the passage of the point of the index finger by forcible upward pressure, giving the impression that the structure above was not involved in the disease. It was therefore determined after consultation with several colleagues, that we had a case of carcinoma of the rectum of a circumscribed nature, which warranted an operation, and extirpation was performed as follows :

The patient was given a whiskey toddy, and a hypodermic injection of morphine, gr. ¼, with atropia gr. $\frac{1}{150}$, was administered, after which he was placed under the anæsthetic influence of the A. C. E. mixture.

Having shaved the surface around the anus and over the sacrum, the parts were thoroughly washed, and all antiseptic precautions were adopted.

An incision of a semi-circular form was made on the posterior border of the anus, so as to extend beyond any of the muscular fibres of the sphincter ani and thus exclude this structure. An incision was then made in the median line from the salient convexity of this curved line to the middle of the sacrum, and carried down to the rectum. The os coccygis was now dissected out and removed, when by dissection and laceration of the cellular tissues around the rectum, it was separated from the adjacent structures. A guide to further proceeding was afforded by the index finger of an assistant being introduced through the anus into the rectum, and ligatures of strong silk were carried around the gut just above the internal sphincter and below the induration. Then, the finger was removed, and the ligatures were drawn tightly and tied, so as to control the blood vessels, when the rectum was divided between them by scissors. The indurated mass was now enucleated with the points of the fingers, until the gut was reached above it, free from any deposition, and it was found that the bowel could be drawn down with little traction. The chain of an ecraseur was passed around the intestine entirely above the indurated and thickened rectum, so as to divide the sound intestinal canal, and thus detach the diseased structure.

The ligature being removed from the lower segment no bleeding occurred, and it was secured by interrupted suture to the upper segment, thus giving an outlet by the natural channel.

Drainage tubes were placed on each side of the intestine and the linear incision closed by suture excepting at the lower end, without stitching the curved line.

There was but little blood lost, and was controlled by the temporary use of forceps with torsion in conjunction with sponges wrung out of a very hot carbolized solution.

The dressing was completed by dusting with iodoform, a thick layer of iodoform gauze, and a compress of absorbent cotton secured by a bandage around the pelvis.

As the vital forces were somewhat depressed, hypodermics of whiskey had been resorted to toward the close of the operation. After rallying from the anæsthetic, the hypodermic of morphia, gr. ¼, with atropia gr. $\frac{1}{150}$, was repeated.

No vomiting ensued, and all food was prohibited for the day, while only milk toast was allowed on the following day.

The patient had a comfortable night, but as it was desirable to keep the bowels from moving the morphia and atropia were used again next evening.

The clinical record for July 9th states that the patient is getting on fairly well, with pulse of 98 and temperature 100°. Upon removing the dressing on account of sero-sanguinolent exudation, the wound presented a good aspect.

The urine has been drawn by catheter, and this is to be continued so as to prevent any straining in the evacuation of the urine. There is no complaint of pain or soreness in the parts involved, and no inclination to evacuate the bowels.

JULY 10.—The report to-day at 12 : 30 P. M. is favorable, pulse being 95 beats and temperature 100°, after a good night's rest. He has taken no opiate this morning, Acet. am. f℥iv., aq. camph. f℥ii., was taken in doses of a dessertspoonful every two hours.

The wound was dressed, and carbolized water thrown in through the drainage tubes. Removed the traction ligature which had been placed in the coats of the upper segment of the intestine, and will remove the drainage tubes to-morrow. He is taking only milk toast thus far as diet.

9 : 30 A. M., JULY 11.—Patient passed a good night under hypodermic, and has pulse of 80 and temperature of 100°. Removed drainage tube this morning, and finding a protruding hemorrhoid, injected 10 per cent. solution of carbolic acid. Wound has a good appearance. Patient is cheerful, with some appetite. No tendency to action of bowels, and less irritation of bladder. Urine has been drawn off with catheter.

JULY 12.—Increase of temperature, being 101°, and pulse 94 beats, with slight tympanites and soreness on percussion over the abdomen. There has been a desire to evacuate the bowels, which, however, has passed off again. There was fæcal matter on the dressings, which evidently escaped from the lower part of the wound and not from the anus, indicating that the stitches had yielded so as to admit of its passage. The tube of a Davidson syringe being inserted at the opening left near the anus, a weak solution of carbolic acid in warm water was thrown up into the wound until it passed out entirely free from stain. With a daily repetition, or oftener if necessary, of this wash, it is hoped that no contamination will occur.

This increase of constitutional disturbance may be temporary, and the appearance of the external wound is satisfactory.

JULY 13.—At 11 A. M. found temperature something less than yesterday, but pulse more frequent, and abdomen still sensitive, yet not tympanitic. Bowels were moved through the wound yesterday evening, requiring change of dressing, and washed out thoroughly then, as also this morning, with carbolized water. He takes light nourishment and is cheerful. Ordered to-day:

℞. Huxam's tinct............f ℥ii.

Tinct. nux vomica.........f ʒi.

Chlorate potash.............ʒi.

Water, q. s................f ℥vi.

Take tablespoonful every four hours as tonic and alterative.

JULY 14.—Patient's condition at noon to-day does not show any aggravation of symptoms since report of yesterday. The temperature is 101¼°; pulse 105 beats; wound presenting good aspect. Fæcal matter still passes by the opening near the anus, and the parts were thoroughly cleansed with the carbolized wash yesterday evening and again this morning, with complete change of dressings. He is anxious to sit up but is not allowed.

JULY 15.—There is nothing in the present condition of the patient to cause any serious apprehension. His temperature at 11 A. M. to-day is 100¼°, being the same as yesterday; but his pulse has risen up to 115 beats, without, however, impairing his appetite or preventing rest at night under the use of the hypodermic (morphine, gr. ¼, artropia, gr. $\frac{1}{150}$). The soreness and tympanites of the abdomen have almost disappeared. Fæces still pass through the wound, but the parts are in good condition.

JULY 16.—A marked change came about rather unexpectedly during the day, ending in collapse. Hypodermics of whiskey and sulphuric ether, along with the external application of mustard and hot bottles to the lower extremities, were resorted to, and quinine and whiskey given internally, but availed nothing. The wound was opened up by removing all the stitches in the cutaneous incision from the anus up to the sacrum, with thorough cleansing of the entire tract and packing with iodoform gauze.

The prostration ended in a fatal result at 11 P. M. If we could have anticipated the septic developments, and opened up the wound forty-eight hours earlier the septicæmia might have been averted.

The question may be raised by some in regard to the advantage of reporting surgical cases which have a fatal termination, and yet these are danger signals which may save the unwary mariner from being wrecked in a voyage upon the same sea. All have realized in their own review of unfavorable cases that a useful lesson was learned, and, perhaps, in this special class of cases no feature of the management is more important than to avoid septic contamination from the contact of the fæces with the fresh cut surface. The absorption being rapid, the same kind of effects are produced as in the perforation of the appendix vermiformis with escape of its fæcal contents, and collapse is the consequence at an early period.

In operating under similar circumstances, the open treatment of the wound commends itself to adoption, even when the walls of the upper and lower segment of the intestine are brought together by suture.

When it was discovered that the fæces escaped through the wound, the removal of all the cutaneous stitches was canvassed; but as there was a free communication from the lower to the upper extremity of the wound, admitting of thorough irrigation, this was not done until it was too late.

Had the wound been exposed and packed with antiseptic gauze, it would have offered a better prospect of success and doubtless would have averted the fatal result. Let others profit by this case, having a good promise and bad ending.

